# Factors associated with obsessive-compulsive symptoms in medical students during the COVID-19 pandemic in Peru: a cross-sectional study

**DOI:** 10.17843/rpmesp.2024.413.13592

**Published:** 2024-09-03

**Authors:** William Alexander Barzola-Farfán, Juan Carlos Ocampo-Zegarra

**Affiliations:** 1 Ayacucho Assistance Network, EsSalud, Ayacucho, Peru Ayacucho Assistance Network EsSalud Ayacucho Peru; 2 Guillermo Almenara Irigoyen National Hospital, Psychiatry Department. Child and Adolescent Psychiatry Service, Lima, Peru. Guillermo Almenara Irigoyen National Hospital Psychiatry Department Child and Adolescent Psychiatry Service Lima Peru

**Keywords:** COVID-19, Medical Students, Obsessive Compulsive Disorder, Mental Health

## Abstract

**Objectives.:**

To determine the prevalence of obsessive-compulsive symptoms among medical students in Peru during the COVID-19 pandemic and its associated factors.

**Materials and methods.:**

Cross-sectional study in 270 medical students from a Peruvian public university. Participants were recruited through non-probability sampling. Obsessive-compulsive symptoms were assessed with the Yale-Brown Obsessive Compulsive Disorder Scale (Y-BOCS). After the descriptive analysis, Poisson regression with robust variance was used to determine the factors associated with probable obsessive compulsive disorder (probable OCD). The crude (PRc) and adjusted (PRa) prevalence ratios were calculated, along with their respective 95% confidence intervals (95% CI).

**Results.:**

The prevalence of obsessive-compulsive symptoms was 13.3% in medical students. During bivariate analysis, students with probable OCD were younger (p=0.044) and had a lower level of knowledge about COVID-19 (p=0.045). The crude model showed a lower prevalence of probable OCD among those with an adequate level of knowledge compared to those with an inadequate level (PR: 0.52, 95% CI: 0.28 to 0.98). However, after adjusting for other variables, none of the described variables were statistically significant.

**Conclusions.:**

One in ten medical students presented clinically significant obsessive-compulsive symptoms. Implementing future interventions is crucial to preserve the mental well-being of this vulnerable population.

## INTRODUCTION

The COVID-19 pandemic has represented a global challenge for health systems since the appearance of the first case in 2019 ^1^. This event has affected the mental health of the general population, particularly in vulnerable groups such as students and health professionals, who have experienced an increase in the incidence of depressive and anxious symptoms ^1^^,^^2^. Likewise, the emergence of stressors derived from the pandemic has led to an increase in the prevalence of obsessive-compulsive symptoms, with a magnitude that has reached up to 20% ^2^^-^^4^.

In medical students, obsessive-compulsive disorder (OCD) has been identified as one of the most frequent disorders and one of the most influenced by the restrictive measures implemented against COVID-19 ^5^^,^^6^. Several factors have been suggested as contributing to the occurrence of these symptoms, including age, level of medical education, and degree of knowledge about the disease ^7^^,^^8^. However, few studies exist on the subject or present inconclusive findings, which may complicate the implementation of specific interventions for this vulnerable population group. Therefore, this study aimed to determine the prevalence of obsessive-compulsive symptoms among Peruvian medical students and to explore their associated factors.

**KEY MESSAGES**

Motivation for the study. The COVID-19 pandemic contributed to the development of mental disorders among medical students, including obsessive-compulsive symptoms. However, evidence on this problem in this population is still limited.

Main findings. One in 10 medical students presented clinically significant obsessive-compulsive symptoms.

Implications. The health crisis has had a negative impact on the mental health of medical students. Therefore, it is crucial to implement future interventions to promote the preservation of their psychological well-being.

## MATERIALS AND METHODS

### Population and study design

A cross-sectional analytical study was conducted in medical students at the Universidad Nacional Mayor de San Marcos (UNMSM). Participants were recruited from all stages of medical training, including the preclinical stage (first to third year), clinical (fourth to sixth year) and medical internship (seventh year). A total of 271 individuals were included by convenience sampling at each stage of medical training. Underage students, those with a previous diagnosis of OCD, those on academic exchange, and those not enrolled at the time of the study were excluded.

### Procedures

The list of 1,336 enrolled medical students and their institutional e-mail addresses were obtained through the academic department of the School of Medicine. Subsequently, between October and November 2022, each student at all stages of medical training was contacted by e-mail. Those students who provided informed consent, after receiving details about the purpose of the study, as well as information about the benefits, risks, and rights associated with participation, were included. Participants completed the questionnaires via a Google form, whose data were coded in Microsoft Excel 365 to ensure confidentiality.

### Variables

The dependent variable of this research was the presence of obsessive-compulsive symptoms, measured by the Yale-Brown Obsessive-Compulsive Disorder Scale (Y-BOCS). This scale consists of a semi-structured questionnaire widely used to estimate the frequency and severity of obsessive-compulsive symptoms, regardless of the specific content of these symptoms ^9^^,^^10^. The instrument consists of 10 items with answer options that are scored between 0 and 4 ^8^^,^^9^. The diagnostic criteria are based on the total score obtained, where a value between 0 and 7 indicates “absence of clinical symptoms”; between 8 and 15, “mild symptoms”; between 16 and 23, “moderate symptoms”; between 24 and 31, “severe symptoms”; and between 32 and 40, “extreme symptoms”. In this study, a cut-off point of 16 or more points on the Y-BOCS scale was established to identify the presence of clinically significant obsessive-compulsive symptoms (probable OCD), as established in previous studies ^8^^,^^11^. This scale can be self-administered or applied remotely and has been validated in Spanish ^12^, with a cultural adaptation for the Peruvian population ^9^.

The level of knowledge about COVID-19 was assessed using the Knowledge of COVID-19 Scale (KNOW-P-COVID-19). This instrument, composed of 9 items, allows the evaluation of essential concepts about the disease, such as the transmission mechanism, mortality and vulnerable subgroups ^13^. This questionnaire, developed in Peru, has a high internal consistency and can be administered to students, health workers and the general population ^13^.

In addition, we collected data on sociodemographic variables, including sex, age, marital status, stage of medical training, place of residence, whether they live alone, and whether they have children. Likewise, personal history of mental disorders, personal history of COVID-19, family history of mental disorders, family history of COVID-19 and deaths due to this disease were assessed. History of COVID-19 was considered as such if the participant had a positive antigen or molecular test result, while history of mental disorder required diagnostic confirmation by a psychiatrist. In addition, were assessed the main source of information about COVID-19 and the hours spent learning about COVID-19.

### Statistical analysis

Descriptive analysis was performed using absolute and relative frequencies for categorical variables. For numerical variables with normal distribution, the mean and standard deviation were used, whereas, for variables with non-normal distribution, we used the median and interquartile range. Inferential analysis was performed to determine the association between the independent variables and probable OCD, using Student’s t-test for normally distributed variables or the chi-square test. The nonparametric Mann-Whitney U test was used for variables with non-normal distribution. In the case of categorical variables with expected values less than 5, Fisher’s exact test was used.

Finally, a multivariate analysis was performed using Poisson regression with robust variance to identify the factors associated with obsessive-compulsive symptoms in medical students. Variables with a p-value<0.20 in the univariate regression were included in the adjusted model. Prevalence ratios (PR) and their 95% confidence intervals were reported for each model. Likewise, all analyses were developed after verifying compliance with the assumptions of linearity in the univariate model and multicollinearity in the adjusted model. The analyses were carried out using the statistical package Stata v.17.0 (Stata Corporation, College Station, Texas, USA), with a statistical significance level set at p < 0.05.

### Ethical considerations

This study was approved by the Research Ethics Committee of the Faculty of Medicine of the Universidad Nacional Mayor de San Marcos. Individuals participated in the study voluntarily, after having provided their informed consent. The methods complied with the principles established in the Declaration of Helsinki.

## RESULTS

After reviewing the database, we excluded one participant due to implausible response options, resulting in a total of 270 participants. The descriptive analysis is presented in Table 1. Of these, 145 (53.7%) were female, the median age was 23 years (interquartile range 20 to 25), and 140 (51.9%) belonged to the preclinical stage of medical training. In addition, 249 (92.2%) resided in Metropolitan Lima, 266 (98.5%) were single, 232 (85.9%) did not live alone, and 266 (98.5%) had no children. Regarding family history, 249 (92.2%) denied having family members with a history of COVID-19, while 183 (67.4%) denied the death of a family member due to this disease. Likewise, 181 (67.0%) reported having no family history of psychiatric disorders.

**Table 1:** Sociodemographic characteristics, history of COVID-19 and mental health in medical students in Peru (n=270).

Variables		n	%
Age (years) ^a^		23 (20-25)	
Sex			
	Male	125	46.3
	Female	145	53.7
Medical training stage			
	Preclinical	140	37.7
	Clinical	102	37.8
	Medical internship	28	10.4
Marital status			
	Single	267	98.9
	Not single	3	1.1
Residence			
	Metropolitan Lima	249	92.2
	Lima Provinces	6	2.3
	Callao	13	4.8
	Other	2	0.7
Lives alone			
	Yes	38	14.1
	No	232	85.9
Has children			
	Yes	4	1.5
	No	266	98.5
Personal history of COVID-19			
	Yes	122	45.2
	No	148	54.8
Personal history of mental disorders			
	Yes	50	18.5
	No	220	81.5
Family history of COVID-19			
	Yes	21	7.8
	No	249	92.2
Family member deceased by COVID-19			
	Yes	88	32.6
	No	182	67.4
Family history of mental disorders			
	Yes	89	33.0
	No	181	67.0
Main source of information on COVID-19			
	Internet (official reports)	179	66.3
	Social networks (Instagram, Facebook, WhatsApp)	48	17.8
	Media (TV, radio)	37	13.7
	Oral (family, friends)	6	2.2
Hours of exposure to media on COVID-19			
	None	61	22.6
	Less than one hour	190	70.4
	Greater than or equal to one hour	19	7.0
Level of knowledge about COVID-19			
	Inadequate (KNOW-P-COVID-19 < 7)	56	20.7
	Adequate (KNOW-P-COVID-19 ≥ 7)	214	79.3
Obsessive-compulsive symptoms			
	Not probable OCD (Y-BOCS < 16)	234	86.7
	probable OCD (Y-BOCS ≥ 16)	36	13.3

a Median and interquartile range (IQR). OCD: Obsessive-compulsive disorder.

The main source of information on COVID-19 was the official reports available on the Internet, which 179 participants (66.3%) relied on. Most students (70.4%) reported spending less than one hour acquiring information about the disease. The level of knowledge about COVID-19 was adequate in 214 (79.3%) of the participants, while 36 (13.3%) had clinically significant obsessive-compulsive symptoms.

The bivariate inferential analysis is presented in Table 2. We identified that the median age was significantly lower among medical students with obsessive-compulsive symptoms (p=0.044). Likewise, the degree of knowledge about COVID-19 was associated with the presence of obsessive-compulsive symptoms (p=0.045), where the highest proportion of students with probable OCD presented poor knowledge about COVID-19.

**Table 2:** Obsessive-compulsive symptoms according to the characteristics of medical students in Peru.

Variables		Obsessive-compulsive symptoms		p-value
		Yes (%)	No (%)	
Total		36 (13.3)	234 (86.7)	
Age (years) ^a^		21 (20-23)	23 (20-25)	0.044*
Sex				
	Male	13 (10.4)	112 (89.6)	0.188
	Female	23 (15.9)	122 (84.1)	
Medical training stage				
	Preclinical	25 (17.9)	115 (82.1)	0.053
	Clinical	10 (9.8)	92 (90.2)	
	Medical internship	1 (3.6)	27 (96.4)	
Marital status ^b^				
	Single	36 (13.5)	231 (86.5)	1,000
	Not single	0 (0)	3 (100)	
Residence ^b^				
	Metropolitan Lima	34 (13.6)	215 (86.4)	0.905
	Lima Provinces	1 (16.7)	5 (83.3)	
	Callao	1 (7.7)	12 (92.3)	
	Other	0 (0)	2 (100)	
Lives alone				
	Yes	5 (13.2)	33 (86.8)	0.973
	No	31 (13.4)	201 (86.6)	
Has children ^b^				
	Yes	1 (25)	3 (75)	0.438
	No	35 (13.2)	231 (86.8)	
Personal history of COVID-19				
	Yes	17 (13.9)	105 (86.1)	0.792
	No	19 (12.8)	129 (87.2)	
Personal history of mental disorders				
	Yes	10 (20)	40 (80)	0.124
	No	26 (11.8)	194 (88.2)	
Family history of COVID-19 ^b^				
	Yes	5 (23.8)	16 (76.2)	0.173
	No	31 (12.5)	218 (87.5)	
Family member deceased by COVID-19				
	Yes	12 (13.6)	76 (86.4)	0.919
	No	24 (13.2)	158 (86.8)	
Family history of mental disorders				
	Yes	15 (16.8)	74 (83.2)	0.233
	No	21 (11.6)	160 (88.4)	
Main source of information on COVID-19 ^b^				
	Internet (official reports)	22 (12.3)	157 (87.7)	0.615
	Social networks (Instagram, Facebook, WhatsApp)	7 (14.6)	41 (85.4)	
	Media (TV, radio)	7 (18.9)	30 (81.1)	
	Oral (family, friends)	0 (0)	6 (100)	
Hours of exposure to media on COVID-19				
	None	10 (16.4)	51 (83.6)	0.703
	Less than one hour	24 (12.6)	166 (87.4)	
	Greater than or equal to one hour	2 (10.5)	17 (89.5)	
Level of knowledge about COVID-19				
	Inadequate	12 (21.4)	44 (78.6)	0.045
	Adequate	24 (11.2)	190 (88.8)	

a Median and interquartile range (IQR). b Fisher’s exact test. ^b^ Mann-Whitney U test.

Finally, the multivariate analysis is presented in Table 3. In the crude model, the prevalence of probable OCD symptoms was found to be 48% lower (95% CI 0.28 to 0.98, p=0.043) among medical students with adequate knowledge about COVID-19 compared to those with inadequate knowledge. In the adjusted regression model, age and family history of COVID-19 were excluded due to multicollinearity. The model was adjusted for sex, stage of medical training, place of residence, personal history of mental disorders, main source of information about COVID-19, and degree of knowledge about COVID-19. However, none of these variables were statistically significant.

**Table 3:** Factors associated with obsessive-compulsive symptoms in medical students in Peru.

Variables		Crude analysis		p-value	Adjusted analysis *		p-value
		PRc	(95% CI)		PRa	(95% CI)	
Sex							
	Male	Ref.			Ref.		
	Female	1.53	(0.81 - 2.89)	0.195	1.33	(0.70 - 2.56)	0.394
Age (years)							
		0.91	(0.82 - 1.01)	0.069	-	-	
Medical training stage							
	Preclinical	Ref.			Ref.		
	Clinical	0.55	(0.81 - 2.89)	0.088	0.58	(0.28 - 1.19)	0.135
	Medical internship	0.20	(0.28 - 1.42)	0.108	0.20	(0.03 - 1.20)	0.078
Marital status							
	Single	Ref.					
	Not single	Not calculable			-	-	-
Residence							
	Metropolitan Lima	Ref.			Ref.		
	Lima Provinces	1.22	(0.20 - 7.53)	0.830	0.96	(0.17 - 5.36)	0.961
	Callao	0.56	(0.08 - 3.81)	0.556	1.13	(0.17 - 7.38)	0.901
	Other	Not calculable			Not calculable		
Lives alone							
	Yes	Ref.					
	No	1.01	(0.42 - 2.45)	0.973	-	-	-
Has children							
	Yes	Ref.					
	No	0.53	(0.09 - 2.96)	0.467	-	-	-
Personal history of COVID-19							
	Yes	Ref.					
	No	0.92	(0.50 - 1.70)	0.792	-	-	-
Personal history of mental disorders							
	No	Ref.			Ref.		
	Yes	1.69	(0.87 - 3.28)	0.120	1.79	(0.91 - 3.53)	0.094
Family history of COVID-19							
	Yes	Ref.					
	No	0.52	(0.23 - 1.20)	0.128	-	-	-
Family member deceased by COVID-19							
	Yes	Ref.					
	No	0.97	(0.57 - 1.84)	0.919	-	-	-
Family history of mental disorders							
	No	Ref.					
	Yes	1.45	(0.79 - 2.68)	0.233	-	-	-
Main source of information on COVID-19							
	Internet (official reports)	Ref.			Ref.		
	Social networks (Instagram, Facebook, WhatsApp)	1.19	(0.54 - 2.61)	0.671	1.11	(0.50 - 2.48)	0.795
	Media (TV, radio)	1.54	(0.71 - 3.34)	0.275	1.32	(0.63 - 2.75)	0.464
	Oral (family, friends)	Not calculable			Not calculable		
Hours of exposure to media on COVID-19							
	None	Ref.					
	Less than one hour	0.77	(0.39 - 1.52)	0.453	-	-	-
	Greater than or equal to one hour	0.64	(0.15 - 2.68)	0.544	-	-	-
Level of knowledge about COVID-19							
	Inadequate	Ref.			Ref.		
	Adequate	0.52	(0.28 - 0.98)	0.043	0.66	(0.35 - 1.23)	0.192

*Adjusted for sex, stage of medical training, residence, history of mental disorders, family history of COVID-19, main source of information about COVID-19, and level of knowledge about COVID-19. Ref.: Reference value.

## DISCUSSION

In this study we found that the prevalence of clinically significant obsessive-compulsive symptoms in medical students was 13.3%. This finding is consistent with the magnitude described in a study conducted in India ^11^, which reported a rate of 14.4% among first- to fourth-year students. However, it was lower than the prevalence reported in a study in the Americas ^14^, which reported a rate of 21.8%. It is possible that factors such as students’ proximity to the front line of health care, uncertainty about the development of future subjects and internships, and the implementation of alternative educational programs may have negatively influenced their mental health and contributed to the development of these symptoms ^14^. Furthermore, differences in reported prevalence rates may be attributed to country-specific contextual factors, such as the phase of the pandemic, contingency measures, and the sociocultural characteristics of the population ^8^^,^^14^. These factors could influence the reaction and degree of resilience of medical students to the health crisis ^8^^,^^14^.

We did not find a significant association between the age of the students and obsessive-compulsive symptoms. This result is inconsistent with a study conducted in Iraq, in which they found that most participants with probable OCD were those in the early years of medical school ^15^. Being young is often associated with less developed coping strategies, which may make it difficult to adapt to stressful situations, such as the COVID-19 pandemic. Furthermore, these coping strategies appear to moderate the impact of stressors related to this public health crisis ^16^. Therefore, younger students may lack the necessary coping skills to deal with stressors such as changes in daily routine, the shift to virtual education, the adoption of preventive measures, and the challenge associated with returning to face-to-face education, which could contribute to the onset of mental disorder ^7^^,^^17^.

Regarding the stage of medical training, no statistically significant association with obsessive-compulsive symptoms was found. This result differs from previous studies that documented a higher rate of probable OCD among students in the first years of medical training ^7^^,^^18^. Being in the early stages of medical training might be associated with an inadequate level of knowledge compared with students in clinical or internship stages, who might have a more informed perception of the impact of COVID-19. Therefore, a lack of knowledge about the disease could lead to inappropriate attitudes and practices ^7^.

Finally, although the crude analysis showed that most students with probable OCD had a low level of COVID-19 knowledge, this association was not found in the adjusted model. These findings differ from previous studies that have shown that higher levels of COVID-19 knowledge were associated with a lower prevalence of mental symptoms in medical students ^8^^,^^17^. Having a solid understanding of the fundamental concepts of the disease could facilitate the development of coping and adaptation skills, promote positive attitudes toward disease prevention, reduce inappropriate practices based on erroneous beliefs, and alleviate fear and anxiety about infection ^17^. This, in turn, could have a positive impact on the mental health of medical students during the COVID-19 pandemic ^17^.

This study has certain limitations. The cross-sectional design could restrict the understanding of the evolution of variables throughout the COVID-19 pandemic. The small sample size could affect the statistical power of the research, and the non-probability sampling method would increase the risk of selection bias. Also, reliance on self-reported data by students could introduce reporting bias and social desirability. Despite these limitations, our study provides valuable information on the prevalence of obsessive-compulsive symptoms among medical students in Peru and highlights the need for diagnostic confirmation and specific interventions in this population.

In conclusion, we identified that at least one in ten medical students presented clinically significant obsessive-compulsive symptoms. However, none of the evaluated variables proved to be statistically significant when analyzing the associated factors. This study highlights the importance of carrying out screening actions to identify these symptoms and provide interventions in the future to address this vulnerable population group.
