# Supplementary material for: Factors associated with obsessive-compulsive symptoms in medical students during the COVID-19 pandemic in Peru: a cross-sectional study
Source: Rev Peru Med Exp Salud Publica. 2024 Sep 3;41(3):259–65. doi: 10.17843/rpmesp.2024.413.13592 (PMC11495934; doi:10.17843/rpmesp.2024.413.13592)
Supplement: Translation

# Translation: Factores asociados con síntomas obsesivo-compulsivos en estudiantes de medicina durante la pandemia por la COVID-19 en Perú: un estudio transversal

**Authors:** William Alexander Barzola-Farfán, Juan Carlos Ocampo-Zegarra

**Affiliations:** 1 Red Asistencial Ayacucho, EsSalud, Ayacucho, Perú; 2 Hospital Nacional Guillermo Almenara Irigoyen, Departamento de Psiquiatría. Servicio de Psiquiatría Infanto Juvenil, Lima, Perú.

**Keywords:** COVID-19, Estudiantes de Medicina, Trastorno Obsesivo Compulsivo, Salud Mental

## Abstract

**Objetivos.:**

Determinar la prevalencia de síntomas obsesivo-compulsivos entre estudiantes de medicina de Perú durante la pandemia por la COVID-19 y sus factores asociados.

**Materiales y métodos.:**

Estudio de corte transversal en 270 estudiantes de medicina de una universidad pública peruana. Los participantes fueron reclutados mediante muestreo no probabilístico. Los síntomas obsesivo-compulsivos fueron evaluados con la Escala de Yale-Brown de Trastorno Obsesivo Compulsivo (Y-BOCS). Luego del análisis descriptivo, se empleó la regresión de Poisson con varianza robusta para determinar los factores asociados a Trastorno Obsesivo Compulsivo probable (TOC probable). Se calcularon las razones de prevalencia crudas (RPc) y ajustadas (RPa), junto con sus respectivos intervalos de confianza del 95% (IC 95%).

**Resultados.:**

La prevalencia de síntomas obsesivo-compulsivos fue de 13,3% en estudiantes de medicina. Durante el análisis bivariado, los estudiantes con TOC probable eran más jóvenes (p=0,044) y tenían un nivel más bajo de conocimiento sobre COVID-19 (p=0,045). En el modelo crudo, se encontró una menor prevalencia de TOC probable entre aquellos con un nivel adecuado de conocimiento en comparación con aquellos con un nivel inadecuado (RPc: 0,52; IC del 95%: 0,28 a 0,98). Sin embargo, después de ajustar por otras variables, ninguna de las variables descritas fue estadísticamente significativa.

**Conclusiones.:**

Uno de cada diez estudiantes de medicina presentó síntomas obsesivo-compulsivos clínicamente significativos. Implementar futuras intervenciones es crucial para preservar el bienestar mental de esta población vulnerable.

## INTRODUCCIÓN

La pandemia de COVID-19 ha representado un desafío global para los sistemas de salud desde la aparición del primer caso en 2019 ^1^. Este evento ha afectado la salud mental de la población en general, especialmente en grupos vulnerables como estudiantes y profesionales de la salud, quienes han experimentado un incremento en la incidencia de síntomas depresivos y ansiosos ^1^^,^^2^. Asimismo, la emergencia de estresores derivados de la pandemia ha llevado a un aumento en la prevalencia de síntomas obsesivo-compulsivos, con una magnitud que ha alcanzado hasta el 20% ^2^^-^^4^.

En los estudiantes de medicina, el Trastorno Obsesivo-Compulsivo (TOC) se ha identificado como uno de los trastornos más frecuentes en esta población y uno de los más influenciados por las medidas restrictivas implementadas contra el COVID-19 ^5^^,^^6^. Diversos factores han sido sugeridos como contribuyentes a la aparición de estos síntomas, incluyendo la edad, el nivel de formación médica y el grado de conocimiento sobre la enfermedad ^7^^,^^8^. No obstante, existen pocos estudios sobre el tema o que presentan hallazgos inconclusos, lo que puede complicar la implementación de intervenciones específicas para este grupo poblacional vulnerable. Por lo tanto, el objetivo del presente estudio fue determinar la prevalencia de síntomas obsesivo-compulsivos entre estudiantes de medicina peruanos y explorar sus factores asociados.

**MENSAJES CLAVE**

Motivación para realizar el estudio. La pandemia de COVID-19 ha contribuido al desarrollo de trastornos mentales entre los estudiantes de medicina, incluyendo síntomas obsesivo-compulsivos. Sin embargo, la evidencia sobre este problema en esta población es aún limitada.

Principales hallazgos. Uno de cada 10 estudiantes de medicina presentó síntomas obsesivo-compulsivos clínicamente significativos.

Implicancias. La crisis sanitaria ha tenido un impacto negativo en la salud mental de los estudiantes de medicina. Por ello, es crucial implementar intervenciones futuras que promuevan la preservación de su bienestar psicológico.

## MATERIALES Y MÉTODOS

### Población y diseño de estudio

Se realizó un estudio analítico de corte transversal en estudiantes de la carrera de Medicina en la Universidad Nacional Mayor de San Marcos (UNMSM). Los participantes fueron reclutados de todas las etapas de la formación médica, incluyendo la etapa preclínica (primer a tercer año), clínica (cuarto a sexto año) e internado médico (séptimo año). Se incluyó a un total de 271 individuos mediante un muestreo por conveniencia en cada etapa de formación médica. Se excluyeron a estudiantes menores de edad, aquellos con diagnóstico previo de TOC, aquellos en situación de intercambio académico y aquellos no matriculados al momento del estudio.

### Procedimientos

Se obtuvo la lista de 1,336 estudiantes de medicina matriculados y sus direcciones de correo electrónico institucionales a través del departamento académico de la Facultad de Medicina. Posteriormente, entre octubre y noviembre de 2022, se contactó a cada estudiante de todas las etapas de formación médica por correo electrónico. Aquellos estudiantes que proporcionaron su consentimiento informado, luego de recibir detalles sobre el objetivo del estudio, así como información sobre los beneficios, riesgos y derechos asociados con la participación, fueron incluidos. Los participantes completaron los cuestionarios a través de un formulario en Google, cuyos datos fueron codificados en Microsoft Excel 365 para garantizar la confidencialidad.

### Variables

La variable dependiente de esta investigación fue la presencia de síntomas obsesivo-compulsivos, medida mediante la Escala de Trastorno Obsesivo-Compulsivo de Yale-Brown (Y-BOCS). Esta escala consiste en un cuestionario semiestructurado ampliamente utilizado para estimar la frecuencia y severidad de los síntomas obsesivo-compulsivos, sin importar el contenido específico de dichos síntomas ^9^^,^^10^. El instrumento consta de 10 ítems con opciones de respuesta que se puntúan entre 0 y 4. (8,9) Los criterios diagnósticos se basan en el puntaje total obtenido, donde un valor entre 0 y 7 indica “ausencia de síntomas clínicos”; entre 8 y 15, “síntomas leves”; entre 16 y 23, “síntomas moderados”; entre 24 y 31, “síntomas severos”; y entre 32 y 40, “síntomas extremos”. En este estudio, se estableció un punto de corte de 16 o más puntos en la escala Y-BOCS para identificar la presencia de síntomas obsesivo-compulsivos clínicamente significativos (TOC probable), conforme a lo establecido en estudios previos ^8^^,^^11^. Esta escala puede ser autoadministrada o aplicada de manera remota y ha sido validada en español ^12^, con una adaptación cultural para la población peruana ^9^.

Se evaluó el nivel de conocimiento sobre el COVID-19 utilizando la Escala de Conocimiento sobre COVID-19 (KNOW-P-COVID-19). Este instrumento, compuesto por 9 ítems, permite la evaluación de conceptos esenciales sobre la enfermedad, como el mecanismo de transmisión, la mortalidad y los subgrupos vulnerables ^13^. Este cuestionario, desarrollado en Perú, presenta una alta consistencia interna y puede ser administrado a estudiantes, trabajadores de la salud y a la población general ^13^.

Adicionalmente, se recopilaron datos sobre variables sociodemográficas, incluyendo sexo, edad, estado civil, etapa de formación médica, lugar de residencia, si viven solos y si tienen hijos. Asimismo, se evaluaron antecedentes personales de trastornos mentales, antecedentes personales de COVID-19, antecedentes familiares de trastornos mentales, antecedentes familiares de COVID-19 y fallecimientos debido a esta enfermedad. Se consideró un antecedente de COVID-19 si el participante había obtenido un resultado positivo en una prueba antigénica o molecular, mientras que un antecedente de trastorno mental requirió la confirmación diagnóstica por parte de un psiquiatra. Además, se evaluaron la principal fuente de información sobre COVID-19 y las horas dedicadas a informarse sobre el tema.

### Análisis estadístico

Se realizó un análisis descriptivo utilizando frecuencias absolutas y relativas para variables categóricas. Para variables numéricas con distribución normal, se emplearon la media y la desviación estándar, mientras que, para variables con distribución no normal, la mediana y el rango intercuartílico. El análisis inferencial se realizó para determinar la asociación entre las variables independientes y el TOC probable, usando la prueba t de Student para variables con distribución normal o la prueba chi-cuadrado. Para variables con distribución no normal, se aplicó la prueba no paramétrica U de Mann-Whitney. En caso las variables categóricas tuvieran valores esperados menores a 5 se utilizó la prueba exacta de Fisher.

Finalmente, se realizó un análisis multivariado mediante regresión de Poisson con varianza robusta para identificar los factores asociados con los síntomas obsesivo-compulsivos en los estudiantes de medicina. Se incluyeron en el modelo ajustado aquellas variables con un valor p<0,20 en la regresión univariada. Se reportaron las razones de prevalencia (RP) y sus intervalos de confianza del 95% para cada modelo. Asimismo, todos los análisis se desarrollaron después de verificar el cumplimiento de los supuestos de linealidad en el modelo univariado y de multicolinealidad en el modelo ajustado. Los análisis se llevaron a cabo utilizando el paquete estadístico Stata v.17.0 (Stata Corporation, College Station, Texas, USA), con un nivel de significancia estadística establecido en p < 0,05.

### Consideraciones éticas

Este estudio fue aprobado por el Comité de Ética en Investigación de la Facultad de Medicina de la Universidad Nacional Mayor de San Marcos. Los individuos participaron del estudio de manera voluntaria, luego de haber proporcionado su consentimiento informado. Los métodos utilizados cumplieron con los principios establecidos en la Declaración de Helsinki.

## RESULTADOS

Después de revisar la base de datos, se excluyó a un participante debido a opciones de respuesta implausibles, resultando en un total de 270 participantes. El análisis descriptivo se presenta en la Tabla 1. De estos, 145 (53,7%) fueron mujeres, la mediana de la edad fue de 23 años (rango intercuartílico de 20 a 25), y 140 (51,9%) pertenecieron a la etapa preclínica de la formación médica. Además, 249 (92,2%) residieron en Lima Metropolitana, 266 (98,5%) fueron solteros, 232 (85,9%) no vivieron solos y 266 (98,5%) no tuvieron hijos. En cuanto a los antecedentes familiares, 249 (92,2%) negaron tener familiares con antecedentes de COVID-19, mientras que 183 (67,4%) negaron el fallecimiento de un familiar debido a esta enfermedad. Asimismo, 181 (67,0%) informaron no tener antecedentes familiares de trastornos psiquiátricos.

**Tabla 1:** Características sociodemográficas, antecedentes de COVID-19 y de salud mental en estudiantes de medicina en Perú (n=270).

Variables		n	%
Edad (años) ^a^		23 (20-25)	
Sexo			
	Masculino	125	46,3
	Femenino	145	53,7
Etapa de formación médica			
	Preclínica	140	37,7
	Clínica	102	37,8
	Internado médico	28	10,4
Estado civil			
	Soltero	267	98,9
	No soltero	3	1,1
Residencia			
	Lima Metropolitana	249	92,2
	Lima Provincias	6	2,3
	Callao	13	4,8
	Otro	2	0,7
Vive solo			
	Sí	38	14,1
	No	232	85,9
Tiene hijos			
	Sí	4	1,5
	No	266	98,5
Antecedente personal de COVID-19			
	Sí	122	45,2
	No	148	54,8
Antecedente personal de trastornos mentales			
	Si	50	18,5
	No	220	81,5
Antecedente familiar de COVID-19			
	Sí	21	7,8
	No	249	92,2
Familiar fallecido por la COVID-19			
	Sí	88	32,6
	No	182	67,4
Antecedente familiar de trastornos mentales			
	Sí	89	33,0
	No	181	67,0
Fuente principal de información sobre COVID-19			
	Internet (reportes oficiales)	179	66,3
	Redes sociales (Instagram, Facebook, WhatsApp)	48	17,8
	Medios de comunicación (TV, radio)	37	13,7
	Oral (familia, amigos)	6	2,2
Horas de exposición a medios de comunicación sobre COVID-19			
	Ninguna	61	22,6
	Menos de una hora	190	70,4
	Mayor o igual a una hora	19	7,0
Nivel de conocimiento sobre COVID-19			
	Inadecuado (KNOW-P-COVID-19 < 7)	56	20,7
	Adecuado (KNOW-P-COVID-19 ≥ 7)	214	79,3
Síntomas obsesivo-compulsivos			
	TOC no probable (Y-BOCS < 16)	234	86,7
	TOC probable (Y-BOCS ≥ 16)	36	13,3

a Mediana y rango intercuartílico (RIQ). TOC: Trastorno Obsesivo-compulsivo.

La principal fuente de información sobre COVID-19 fueron los informes oficiales disponibles en internet, en los que confiaron 179 participantes (66,3%). La mayoría de estudiantes (70,4%) reportaron dedicar menos de una hora a la adquisición de información sobre la enfermedad. El nivel de conocimiento sobre COVID-19 fue adecuado en 214 (79,3%) de los participantes, mientras que 36 (13,3%) presentaron síntomas obsesivo-compulsivos clínicamente significativos.

El análisis inferencial bivariado se presenta en la Tabla 2. Se identificó que la mediana de edad fue significativamente menor entre los estudiantes de medicina con síntomas obsesivo-compulsivos (p=0,044). Asimismo, el grado de conocimiento sobre COVID-19 estuvo asociado con la presencia de síntomas obsesivo-compulsivos (p=0,045), donde la mayor proporción de estudiantes con TOC probable presentó un conocimiento deficiente sobre la COVID-19.

**Tabla 2:** Síntomas obsesivo-compulsivos de acuerdo con las características de los estudiantes de medicina en Perú.

Variables		Síntomas obsesivo-compulsivos		Valor de p
		Sí (%)	No (%)	
Total		36 (13,3)	234 (86,7)	
Edad (años) ^a^		21 (20-23)	23 (20-25)	0,044*
Sexo				
	Masculino	13 (10,4)	112 (89,6)	0,188
	Femenino	23 (15,9)	122 (84,1)	
Etapa de formación médica				
	Preclínica	25 (17,9)	115 (82,1)	0,053
	Clínica	10 (9,8)	92 (90,2)	
	Internado médico	1 (3,6)	27 (96,4)	
Estado civil ^b^				
	Soltero	36 (13,5)	231 (86,5)	1,000
	No soltero	0 (0)	3 (100)	
Residencia ^b^				
	Lima Metropolitana	34 (13,6)	215 (86,4)	0,905
	Lima Provincias	1 (16,7)	5 (83,3)	
	Callao	1 (7,7)	12 (92,3)	
	Otro	0 (0)	2 (100)	
Vive solo				
	Sí	5 (13,2)	33 (86,8)	0,973
	No	31 (13,4)	201 (86,6)	
Tiene hijos ^b^				
	Sí	1 (25)	3 (75)	0,438
	No	35 (13,2)	231 (86,8)	
Antecedente personal de COVID-19				
	Sí	17 (13,9)	105 (86,1)	0,792
	No	19 (12,8)	129 (87,2)	
Antecedente personal de trastornos mentales				
	Sí	10 (20)	40 (80)	0,124
	No	26 (11,8)	194 (88,2)	
Antecedente familiar de COVID-19 ^b^				
	Sí	5 (23,8)	16 (76,2)	0,173
	No	31 (12,5)	218 (87,5)	
Familiar fallecido por la COVID-19				
	Sí	12 (13,6)	76 (86,4)	0,919
	No	24 (13,2)	158 (86,8)	
Antecedente familiar de trastornos mentales				
	Sí	15 (16,8)	74 (83,2)	0,233
	No	21 (11,6)	160 (88,4)	
Fuente principal de información sobre COVID-19 ^b^				
	Internet (reportes oficiales)	22 (12,3)	157 (87,7)	0,615
	Redes sociales (Instagram, Facebook, WhatsApp)	7 (14,6)	41 (85,4)	
	Medios de comunicación (TV, radio)	7 (18,9)	30 (81,1)	
	Oral (familia, amigos)	0 (0)	6 (100)	
Horas de exposición a medios de comunicación sobre COVID-19				
	Ninguna	10 (16,4)	51 (83,6)	0,703
	Menos de una hora	24 (12,6)	166 (87,4)	
	Mayor o igual a una hora	2 (10,5)	17 (89,5)	
Nivel de conocimiento sobre COVID-19				
	Inadecuado	12 (21,4)	44 (78,6)	0,045
	Adecuado	24 (11,2)	190 (88,8)	

a Mediana y rango intercuartílico (RIQ). ^b^ Prueba exacta de Fisher. *Prueba U de Mann-Whitney.

Finalmente, el análisis multivariado se presenta en la Tabla 3. En el modelo crudo, se encontró que la prevalencia de síntomas probables de TOC fue un 48% menor (IC 95%: 0,28 a 0,98, p=0,043) entre los estudiantes de medicina con un conocimiento adecuado sobre COVID-19 en comparación con aquellos con un conocimiento inadecuado. En el modelo de regresión ajustado, se excluyeron la edad y los antecedentes familiares de COVID-19 debido a la multicolinealidad. El modelo se ajustó para sexo, etapa de formación médica, lugar de residencia, antecedentes personales de trastornos mentales, principal fuente de información sobre COVID-19 y grado de conocimiento sobre COVID-19. Sin embargo, ninguna de estas variables resultó estadísticamente significativa.

**Tabla 3:** Factores asociados con síntomas obsesivo-compulsivos en estudiantes de medicina en Perú.

Variables		Análisis crudo		Valor de p	Análisis ajustado*		Valor de p
		RPc	(IC 95%)		RPa	(IC 95%)	
Sexo							
	Masculino		Ref.			Ref.	
	Femenino	1,53	(0,81 a 2,89)	0,195	1,33	(0,70 a 2,56)	0,394
Edad (años)							
		0,91	(0,82 a 1,01)	0,069	-	-	
Etapa de formación médica							
	Preclínica		Ref.			Ref.	
	Clínica	0,55	(0,81 a 2,89)	0,088	0,58	(0,28 a 1,19)	0,135
	Internado médico	0,20	(0,28 a 1,42)	0,108	0,20	(0,03 a 1,20)	0,078
Estado civil							
	Soltero		Ref.				
	No soltero		No calculable		-	-	-
Residencia							
	Lima Metropolitana		Ref.			Ref.	
	Lima Provincias	1,22	(0,20 a 7,53)	0,830	0,96	(0,17 a 5,36)	0,961
	Callao	0,56	(0,08 a 3,81)	0,556	1,13	(0,17 a 7,38)	0,901
	Otro		No calculable			No calculable	
Vive solo							
	Sí		Ref.				
	No	1,01	(0,42 a 2,45)	0,973	-	-	-
Tiene hijos							
	Sí		Ref.				
	No	0,53	(0,09 a 2,96)	0,467	-	-	-
Antecedente personal de COVID-19							
	Sí		Ref.				
	No	0,92	(0,50 a 1,70)	0,792	-	-	-
Antecedente personal de trastornos mentales							
	No		Ref.			Ref.	
	Sí	1,69	(0,87 a 3,28)	0,120	1,79	(0,91 a 3,53)	0,094
Antecedente familiar de COVID-19							
	Sí		Ref.				
	No	0,52	(0,23 a 1,20)	0,128	-	-	-
Familiar fallecido por la COVID-19							
	Sí		Ref.				
	No	0,97	(0,57 a 1,84)	0,919	-	-	-
Antecedente familiar de trastornos mentales							
	No		Ref.				
	Sí	1,45	(0,79 a 2,68)	0,233	-	-	-
Fuente principal de información sobre COVID-19							
	Internet (reportes oficiales)		Ref.			Ref.	
	Redes sociales (Instagram, Facebook, WhatsApp)	1,19	(0,54 a 2,61)	0,671	1,11	(0,50 a 2,48)	0,795
	Medios de comunicación (TV, radio)	1,54	(0,71 a 3,34)	0,275	1,32	(0,63 a 2,75)	0,464
	Oral (familia, amigos)		No calculable			No calculable	
Horas de exposición a medios de comunicación sobre COVID-19							
	Ninguna		Ref.				
	Menos de una hora	0,77	(0,39 a 1,52)	0,453	-	-	-
	Mayor o igual a una hora	0,64	(0,15 a 2,68)	0,544	-	-	-
Nivel de conocimiento sobre COVID-19							
	Inadecuado		Ref.			Ref.	
	Adecuado	0,52	(0,28 a 0,98)	0,043	0,66	(0,35 a 1,23)	0,192

*Ajustado por sexo, etapa de formación médica, residencia, antecedente de trastornos mentales, antecedente familiar de COVID-19, fuente principal de información sobre COVID-19, y nivel de conocimiento sobre COVID-19. Ref.: Valor de referencia.

## DISCUSIÓN

En el presente estudio, los investigadores encontraron que la prevalencia de síntomas obsesivo-compulsivos clínicamente significativos en estudiantes de medicina fue del 13,3%. Este hallazgo es consistente con la magnitud descrita en un estudio realizado en India ^11^, que reportó una tasa del 14,4% entre estudiantes de primer a cuarto año. Sin embargo, fue menor que la prevalencia reportada en un estudio a nivel del continente americano ^14^, donde se informó una tasa del 21,8%. Es posible que factores como la proximidad de los estudiantes a la primera línea de atención sanitaria, la incertidumbre sobre el desarrollo de futuras materias y prácticas, y la implementación de programas educativos alternativos hayan influido negativamente en su salud mental y contribuido al desarrollo de estos síntomas ^14^. Asimismo, las diferencias en las tasas de prevalencia reportadas pueden ser atribuidas a factores contextuales específicos de cada país, como la fase de la pandemia, las medidas de contingencia adoptadas y las características socioculturales de la población ^8^^,^^14^. Estos factores podrían influir en la reacción y el grado de resiliencia de los estudiantes de medicina frente a la crisis sanitaria ^8^^,^^14^.

Los autores no encontraron una asociación significativa entre la edad de los estudiantes y los síntomas obsesivo-compulsivos. Este resultado es inconsistente con un estudio realizado en Irak, donde encontró que la mayoría de los participantes con TOC probable pertenecieron a los primeros años de la carrera de medicina ^15^. Ser joven a menudo se asocia con estrategias de afrontamiento menos desarrolladas, lo que puede dificultar la adaptación a situaciones estresantes, como la pandemia de COVID-19. Además, estas estrategias de afrontamiento parecen moderar el impacto de los estresores relacionados con esta crisis de salud pública ^16^. Por lo tanto, los estudiantes más jóvenes podrían carecer de las habilidades necesarias para enfrentar estresores como los cambios en la rutina diaria, el cambio a la educación virtual, la adopción de medidas preventivas y el desafío asociado con el regreso a la educación presencial, lo que podría contribuir al inicio de un trastorno mental ^7^^,^^17^.

En cuanto a la etapa de la formación médica, no se encontró una asociación estadísticamente significativa con los síntomas obsesivo-compulsivos. Este resultado difiere de estudios previos que documentaron una mayor tasa de TOC probable entre los estudiantes pertenecientes a los primeros años de la carrera de medicina ^7^^,^^18^. Cursar las etapas iniciales de la formación médica podría asociarse con un nivel de conocimiento inadecuado en comparación con los estudiantes en etapas clínicas o de internado, quienes podrían tener una percepción más informada sobre el impacto del COVID-19. Por lo tanto, una falta de conocimiento sobre la enfermedad podría llevar a actitudes y prácticas inapropiadas ^7^.

En cuanto a la etapa de la formación médica, no se encontró una asociación estadísticamente significativa con los síntomas obsesivo-compulsivos. Este resultado difiere de estudios previos que documentaron una mayor tasa de TOC probable entre los estudiantes pertenecientes a los primeros años de la carrera de medicina ^7^^,^^18^. Cursar las etapas iniciales de la formación médica podría asociarse con un nivel de conocimiento inadecuado en comparación con los estudiantes en etapas clínicas o de internado, quienes podrían tener una percepción más informada sobre el impacto de la COVID-19. Por lo tanto, una falta de conocimiento sobre la enfermedad podría conllevar a actitudes y prácticas inapropiadas ^7^.

Finalmente, si bien el análisis crudo mostró que la mayoría de los estudiantes con TOC probable tuvieron un bajo nivel de conocimiento sobre COVID-19, esta asociación no se observó en el modelo ajustado. Estos hallazgos difieren de estudios previos que han mostrado que niveles más altos de conocimiento sobre COVID-19 estuvieron asociados con una menor prevalencia de síntomas mentales en estudiantes de medicina ^8^^,^^17^.

Contar con una comprensión sólida de los conceptos fundamentales de la enfermedad podría facilitar el desarrollo de habilidades de afrontamiento y adaptación, promover actitudes positivas hacia la prevención de la enfermedad, reducir prácticas inapropiadas basadas en creencias erróneas, y aliviar el miedo y la ansiedad sobre la infección ^17^. Esto, a su vez, podría tener un impacto positivo en la salud mental de los estudiantes de medicina durante la pandemia por la COVID-19 ^17^.

Este estudio presenta ciertas limitaciones. El diseño transversal podría restringir la comprensión de la evolución de las variables a lo largo de la pandemia por la COVID-19. El tamaño reducido de la muestra podría afectar el poder estadístico de la investigación, y el método de muestreo no probabilístico aumentaría el riesgo de sesgo de selección. Asimismo, la confianza en datos auto reportados por parte de los estudiantes podría introducir sesgos de reporte y deseabilidad social. A pesar de estas limitaciones, el estudio ofrece información valiosa sobre la prevalencia de síntomas obsesivo-compulsivos entre estudiantes de medicina en Perú y resalta la necesidad de la confirmación diagnóstica e intervenciones específicas en esta población.

En conclusión, se identificó que al menos uno de cada diez estudiantes de medicina presentó síntomas obsesivo-compulsivos clínicamente significativos. No obstante, ninguna de las variables evaluadas resultó ser estadísticamente significativa al analizar los factores asociados. Este estudio subraya la importancia de llevar a cabo acciones de tamizaje para identificar estos síntomas y proporcionar intervenciones en el futuro para abordar a este grupo poblacional vulnerable.
